# Supplementary material for: Species-level verification of Phascolarctobacterium association with colorectal cancer
Source: mSystems. 2024 Sep 17;9(10):e00734-24. doi: 10.1128/msystems.00734-24 (PMC11494908; doi:10.1128/msystems.00734-24)
Supplement: Supplemental figures — Figures S1 and S2. [file msystems.00734-24-s0001.pdf]

Species-level verification of *Phascolarctobacterium* association to colorectal cancer

Cecilie Bucher-Johannessen, Thulasika Senthakumaran, Ekaterina Avershina, Einar Birkeland, Geir Hoff, Vahid Bemanian, Hege Tunsjø, Trine B Rounge

Supplementary figures

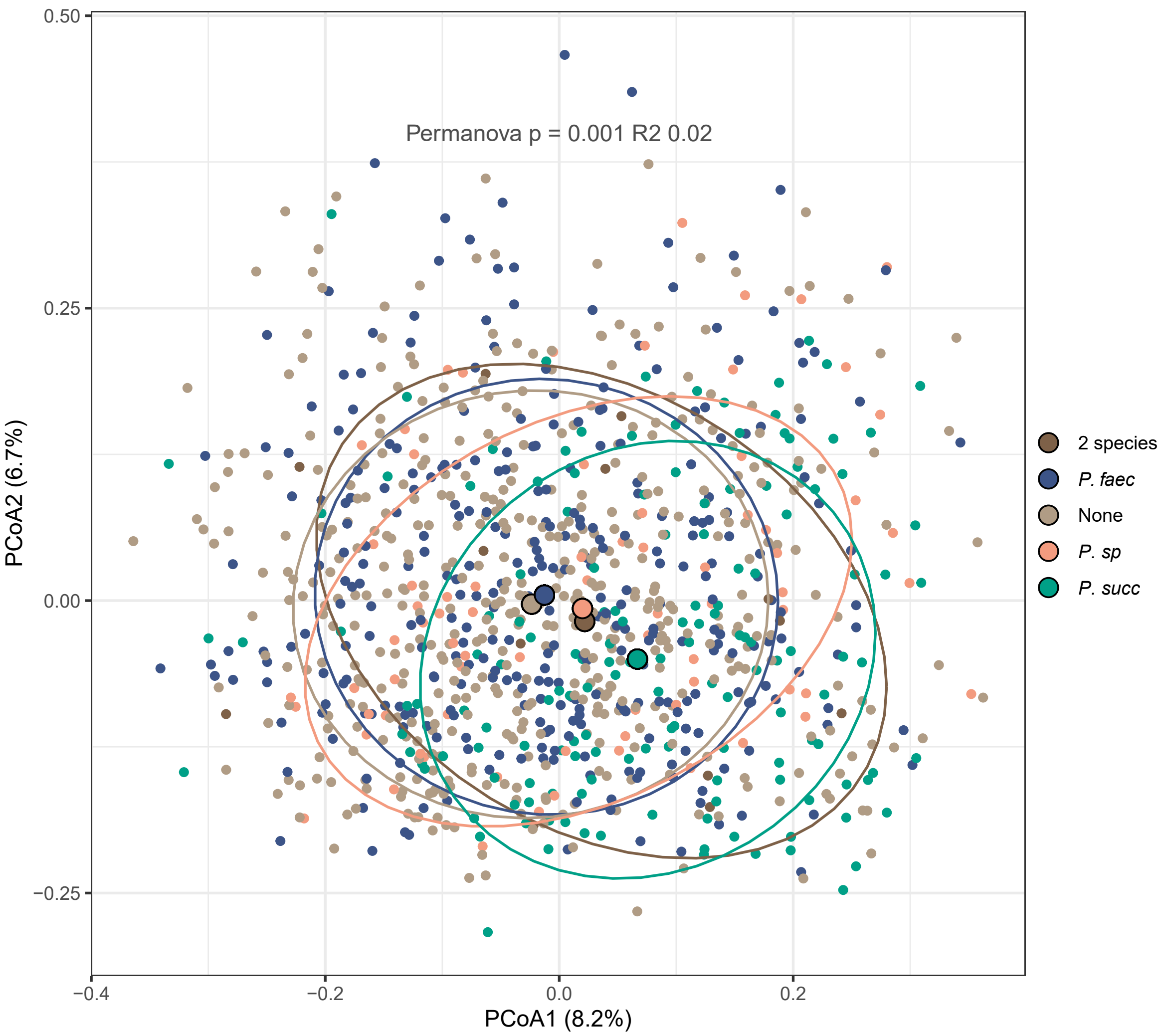

Figure S1: PCoA plot of microbiome composition in CRCbiome samples. Groups are defined as those samples that have reads from only *P. succinatutens*, *P.* sp 377, *P. faecium*, two *Phascolarctobacterium* or no *Phascolarctobacterium*. PERMANOVA results showed a significant difference between groups ($p<0.001$) with an R^2 of 0.02.

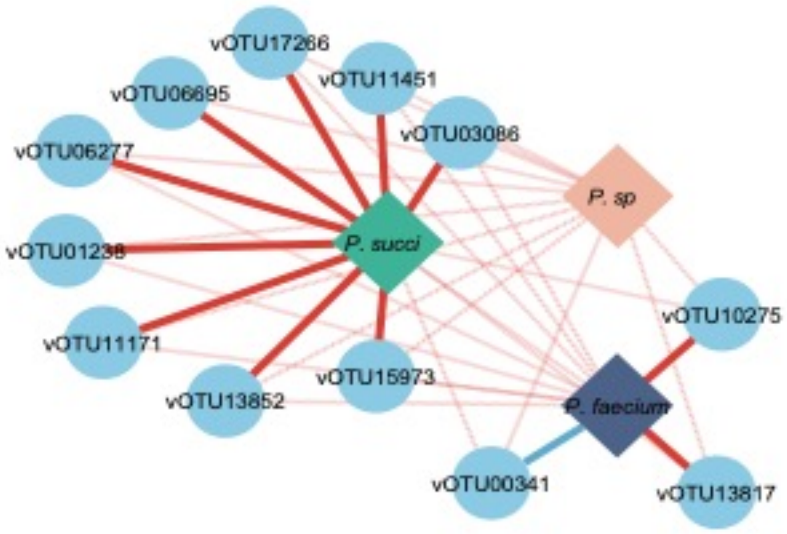

Figure S2: vOTU correlation plot. Presence/absence-based co-occurrence network between *Phascolarctobacterium* species and viruses in the CRCbiome dataset. Solid lines indicate significant correlations (FDR-corrected $p < 0.05$), whereas shaded lines indicate non-significant correlations. Only vOTUs with a significant correlation to one of the *Phascolarctobacterium* MAGs are depicted. Red: tendency to co-exclusion; Blue: tendency to co-existence. The direction of the correlation was defined based on the difference between the observed “yes/yes" and the expected “yes/yes”.
